# Unlocking the potential of circulating small extracellular vesicles in neurodegenerative disease through targeted biomarkers and advancements in biosensing

**DOI:** 10.37349/ebmx.2024.00008

**Published:** 2024-04-24

**Authors:** Saqer Al Abdullah, Ivy Cocklereece, Kristen Dellinger

**Affiliations:** 1 Department of Nanoengineering, Joint School of Nanoscience and Nanoengineering, North Carolina A&T State University, Greensboro, NC 27401, USA; 2 Department of Nanoscience, Joint School of Nanoscience and Nanoengineering, University of North Carolina at Greensboro, Greensboro, NC 27401, USA

**Keywords:** Exosomes, biosensing, Alzheimer’s disease, Parkinson’s disease

## Abstract

Neurodegenerative diseases (NDDs) gradually affect neurons impacting both their function and structure, and they afflict millions worldwide. Detecting these conditions before symptoms arise is crucial for better prognosis and duality of life, given that the disease processes often begin years earlier. Yet, reliable and affordable methods to diagnose NDDs in these stages are currently lacking. There’s a growing interest in using circulating extracellular vesicles (EVs), like small EVs (sEVs) also known as exosomes, as potential sources of markers for screening, diagnosing, and monitoring NDDs. This interest stems from evidence showing that these EVs can carry brain pathological proteins implicated in NDD pathology, and they can even traverse the blood-brain barrier. This review focuses on the creation of EVs, particularly sEVs with a size of less than 200 nanometers, methods for isolating sEVs, and recent advancements in biosensor development to detect NDD-related markers found in sEVs. Furthermore, it explores the potential of sEVs in diagnosing four major NDDs: Alzheimer’s disease (AD), Parkinson’s disease (PD), Huntington’s disease (HD), and multiple system atrophy (MSA).

## Introduction

Neurodegenerative diseases (NDDs) pose a significant burden on patients and their families, with more than 50 million people living with dementia in 2016, and the number of cases is expected to double by 2050 [1]. While multifactorial and varied with respect to onset and manifestation, NDDs are generally characterized by gradual neuronal death in the central nervous system (CNS) that affects the brain’s functions and several body activities, such as movement, speaking, and breathing. NDDs encompass a group of life-threatening illnesses that include Alzheimer’s disease (AD), Parkinson’s disease (PD), Huntington’s disease (HD), multiple system atrophy (MSA), progressive supranuclear palsy, and spinocerebellar ataxias. A signature cause of NDDs is the accumulation of specific misfolded protein that leads to neuronal loss in different regions of the brain [2]. Currently, NDDs are most commonly diagnosed upon clinical presentation of symptoms, which are corroborated with multifactorial panels, including clinical history, cognitive tests, medical examinations, imaging, and cerebrospinal fluid (CSF) biomarker analysis. Due to the limitations of these current diagnostic techniques being expensive, invasive, and unable to detect NDDs in their early stages, several efforts have been made to discover new diagnostic methods and target biomarkers that are more accurate, cost-effective, non-invasive, and capable of detecting NDDs in early stages to facilitate more effective interventions [2, 3].

**Figure 1.**
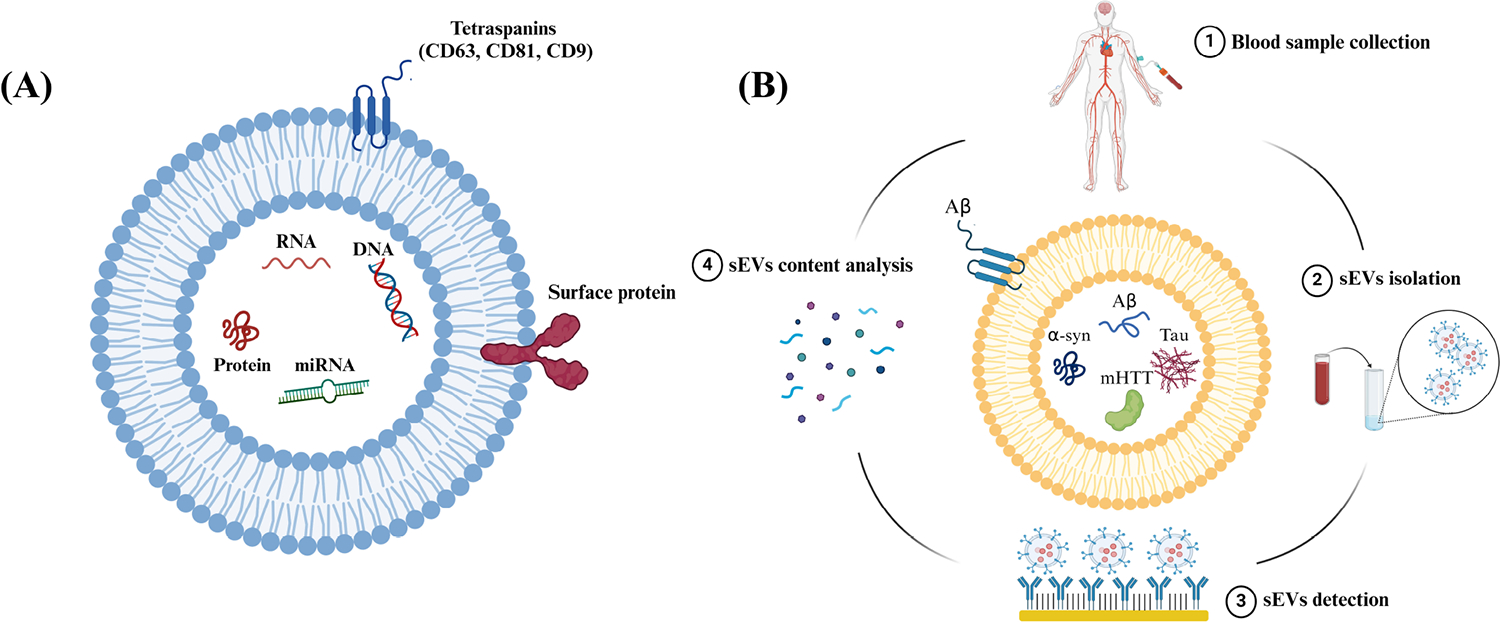
Schematic representation showing sEVs structure and steps included in the analysis of sEVs content. (A) Schematic representation showing the structure and common components of sEVs. sEVs are composed of a phospholipid bilayer embedded with surface proteins and tetraspanins. sEVs encapsulate biological molecules, such as DNA, RNA, miRNA, and proteins. CD: cluster of differentiation; (B) schematic representation showing the steps to use peripheral circulating sEVs as sources of biomarkers for NDDs. Created in BioRender (https://www.biorender.com/)

Extracellular vesicles (EVs), encompass a group of lipid-bound structures, that include microvesicles or ectosomes (100–1000 nm) and small EVs (sEVs) or also known as exosomes (< 200 nm), that contain and transport several molecules, including nucleic acids and surface-specific embedded proteins [4]. EVs were considered a waste pathway for cells to eliminate unwanted proteins and cellular components in the past [4]. However, recent evidence has shown that these particles perform variable functions and are considered key communication tools among cells [5–8]. This is because they act as vehicles that can travel between cells carrying DNA, RNA, microRNA (miRNA), and proteins representing their cell of origin, see Figure 1A [4, 8]. sEVs are released from every cell in the body and detected in various body fluids, including urine, saliva, and blood [9–12]. In the CNS, sEVs play a significant role in several physiological processes, such as intercellular communication and synaptic plasticity [13]. Moreover, it has been found that sEVs also play a role in the progression of brain diseases *via* the transfer of pathological proteins between cells, such as amyloid-β (Aβ) protein and tau in AD [14], α-synuclein (α-syn) in PD and MSA [15], and mutant huntingtin protein (mHTT) in HD [16]. Interestingly, it has been discovered that sEVs carry these proteins among cells in the brain and can also cross the blood-brain barrier (BBB), carrying their content from the brain into the peripheral blood system [17, 18]. An overview of the potential approach to utilizing sEVs derived from systemic circulation in conjunction with miniaturized screening or diagnostic platforms for NDDs is shown in Figure 1B.

These recent revelations have sparked interest in employing brain-derived sEVs from the peripheral circulation to overcome the limitations of conventional NDDs diagnostic techniques, such as neuroimaging and CSF analysis. This is because brain-derived sEVs are accessible in biological fluids that can be easily collected, such as blood, and their contents have the potential to reflect the pathophysiological progression of disease in the brain [12]. This review will briefly discuss sEVs biogenesis, approaches to isolating sEVs, and recent biosensor designs to detect NDD-relevant sEVs derived from the peripheral circulation. This review will additionally focus on recent progress in the application of brain-derived sEVs proteins for diagnosing four major NDDs, namely AD, PD, HD, and MSA. In this paper, the authors attempt to comprehensively review the literature on EVs, in particular, sEVs, in the last 10 years from their characteristics to their potential applications in the diagnosis and prognosis of the aforementioned NDDs. An extensive search was conducted using PubMed, Google Scholar, and Web of Science databases with the keywords ‘exosomes’, ‘exosomal’, ‘EVs’, or ‘sEVs’, combined with ‘biomarker’, ‘NDDs’, ‘AD’, ‘PD’, ‘HD’, or ‘MSA’.

## Mechanisms of sEVs biogenesis

Understanding the process and factors that influence sEVs biogenesis pathways in the CNS could help researchers engineer sEVs to target specific pathways, deliver cargo (e.g., therapeutic peptides or small molecules), and develop interventions to influence the pathogenesis of various diseases. Moreover, this could open the floodgates of possibilities to utilize sEVs as novel repositories for biomarkers to diagnose multifactorial NDDs, such as AD and PD.

**Figure 2.**
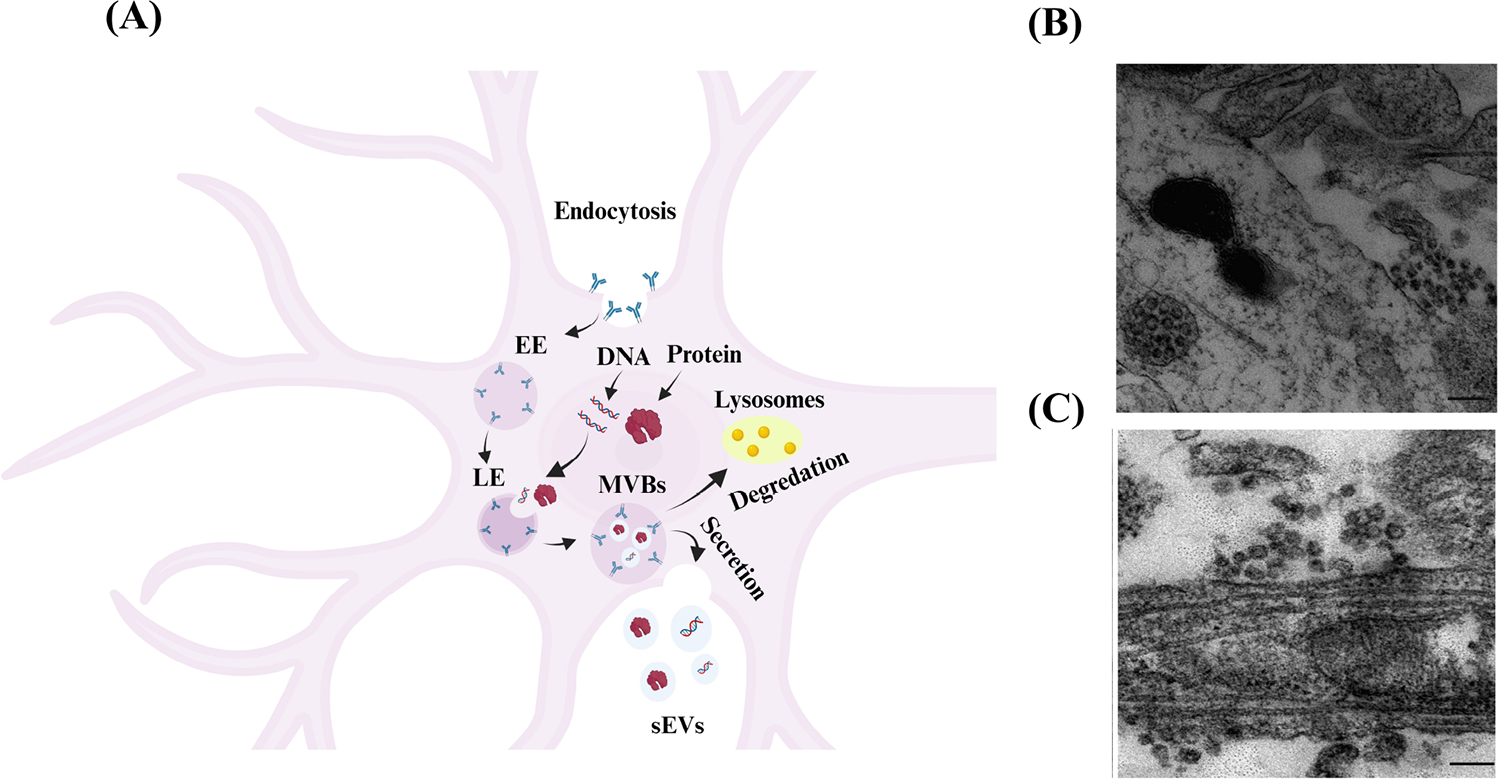
Biogenesis and secretion of sEVs from neuronal cells. (A) Schematic representation showing the biogenesis of as sEVs in neuronal cells [19]. Budding of the cellular plasma membrane leads to the formation of EE vesicles. EEs mature and turn into LEs. Cellular components, such as DNA, RNA, and protein, invaginate into LEs and form ILVs that result in the formation of MVB vesicles. MVBs are either fused to lysosomes and degraded by lysosomal enzymes or with the cell’s surface and released as sEVs. Created in BioRender; (B) electron microscope image of cortical neurons shows MVB clusters close to the plasma membrane of the soma [20]; (C) electron microscope image of cortical neurons shows MVB clusters close to the plasma membrane of dendrites [20]. The scale bar for Figure 2B and 2C is 200 nm *Note*. Figure 2A was adapted with permission from “Exosomes and other EVs in neural cells and NDDs,” by Janas AM, Sapoń K, Janas T, Stowell MH, Janas T. Biochim Biophys Acta. 2016;1858:1139–51 (https://doi.org/10.1016/j.bbamem.2016.02.011). © 2016 Elsevier B.V.; Figure 2B and 2C were reprinted with permission from “Release of exosomes from differentiated neurons and its regulation by synaptic glutamatergic activity,” by Lachenal G, Pernet-Gallay K, Chivet M, Hemming FJ, Belly A, Bodon G, et al. Mol Cell Neurosci. 2011;46:409–18 (https://doi.org/10.1016/j.mcn.2010.11.004). © 2010 Elsevier Inc.

As shown in Figure 2A, the most common pathway of sEVs production of neuronal cells consists of three steps: (I) budding and pinching of the plasma membrane occurs on the surface of the cell membrane, which influences the production of early endosome (EE) vesicles that mature into late endosomes (LEs) [4]. (II) Intracellular molecules, such as DNA and proteins, invaginate into the LEs and form smaller vesicles inside the endosomes, called intraluminal vesicles (ILVs) [4]. After the ILVs are formed, LEs turn into multivesicular body (MVB) vesicles [4]. (III) MVBs are either degraded by lysosomes or fuse with the cell’s plasma membrane and are released into the extracellular space. These released vesicles, with a size of less than 200 nm, are known as ‘sEVs’ [4].

sEVs are secreted from numerous cells in the CNS, including neurons [21], oligodendrocytes [22], and astrocytes [23], carrying their contents to facilitate cell-to-cell communication. Several factors regulate the fusing of MVBs to the plasma membrane and the release of their content (including sEVs) into the extracellular space. For one, the release of sEVs from neuronal cells may be triggered by cell depolarization or other mechanisms that shift neuronal cells into an excitatory state, such as Ca^2+^ influx and gamma-aminobutyric acid receptor antagonists. Lachenal et al. [20] observed sEVs secretion in two regions of the neuronal cell, namely the soma and dendritic shaft, as shown in Figure 2B and 2C. Further, they found that inducing neuronal depolarization enhanced the secretion of sEVs from mature neurons and that glutamatergic activity plays a crucial role in regulating the secretion of sEVs from these cells [20].

sEVs secretion from oligodendrocytes, whose primary function is to myelinate axons in the brain, has been shown to be influenced by Ca^2+^ flux into the cells [22]. Frühbeis et al. [24] found that glutamate neurotransmitters released from neurons enhance Ca^2+^ influx into oligodendrocytes *via N*-methyl-D-aspartate and alpha-amino-3-hydroxy-5-methyl-4-isoxazole-propionic acid glutamate receptors leading to an enhancement in the secretion of sEVs from oligodendrocytes. The oligodendrocyte-secreted sEVs return and enter neurons carrying certain proteins, such as proteolipid protein, that contribute to the myelination process of neuronal axons [24, 25].

As discussed further in the following sections, sEVs may mediate the propagation of pathological proteins between cells in several NDDs, including AD, PD, HD, and MSA. Thus, it has been suggested that controlling the formation and secretion of sEVs might be a novel therapeutic strategy for treating NDDs [26, 27]. However, controlling their formation and secretion is highly challenging since sEVs biogenesis is a complex and multifactorial process. Managing the formation and secretion of sEVs may necessitate multitarget compounds that can selectively block interrelated sEVs secretion factors. Thus, studying the factors that control sEVs biogenesis and release in the CNS might help characterize the pathology of NDDs and the relationship between sEVs secretion and the progression of these diseases. Moreover, such studies might accelerate the discovery of new NDD biomarkers.

Since sEVs are secreted from all cells in the brain and can cross the BBB carrying pathogenic proteins from the brain into the peripheral blood [17, 18], peripheral circulating CNS-derived sEVs may provide a repository of molecular information that can be analyzed to diagnose NDDs. However, to progress effectively in this field, a few fundamental challenges must be overcome. One of the most critical obstacles is creating efficient, reliable, and cost-effective methods for isolating sEVs from blood. Thus, the current state of sEVs isolation methods of brain-derived sEVs from the peripheral circulation is reviewed and discussed in the following section.

## Refining the process: techniques and innovations in isolating EVs

To fully realize the potential of sEVs as diagnostic tools for NDDs, isolation methods that can differentiate brain-derived sEVs, such as neuronal sEVs, astrocytic sEVs, and oligodendroglial sEVs, from other small EV subpopulations in the peripheral circulation are needed. Since the concentration of brain-derived sEVs in blood circulation is very low, detecting brain-derived sEVs from blood samples and analyzing their content is highly challenging [28]. Thus, developing practical isolation methods will improve accuracy, detection limits, and the effectiveness and reliability of sEVs detection technologies. The following discussion focuses on sEVs isolation methods that have been used to isolate brain-derived sEVs from the peripheral circulation.

### Ultracentrifugation

Ultracentrifugation is widely used to isolate sEVs from other components of biological samples, such as apoptotic bodies, based on differences in the sedimentation rate. Ultracentrifuges rotate at high speeds (on the order of 100,000 to 120,000 g) and can be categorized into two main groups: analytical ultracentrifugation (AUC) and preparative centrifugation [29]. AUCs are high-speed ultracentrifuges equipped with optic systems to monitor the fractionation process of particles and determine properties, such as sedimentation rate in solution, shape, and mass [29]. The preparative centrifugation procedure is widely used to purify biological particles, including cells, viruses, nucleic acids, proteins, and sEVs. The basic principle of this approach is to isolate small particles from large particles by upregulating the applied centrifugal force and duration of centrifugation stepwise [29]. As the centrifugation field proceeds, particles of the sample are settled throughout the centrifuge tube based on their sedimentation rate [29]. However, isolating sEVs *via* ultracentrifugation is time-consuming, requires several hours, and is both costly and not widely available, especially in clinical labs. Another drawback of this method is that it can produce low yields [30]. Ultracentrifugation has been utilized to isolate sEVs from plasma samples of individuals with PD and healthy controls [31]. The authors used a 2-step ultracentrifugation method, with a speed of 180,000 *g* for 3 h at 4°C to isolate sEVs from sEV-poor plasma samples [31].

### Size-based isolation

Two isolation techniques are widely used to separate EVs based on size: differential filtration and size exclusion chromatography (SEC). First, differential filtration is used to isolate sEVs from serum and cell culture media based on size and molecular weight [32]. This technique comprises of multiple filtration steps: (I) a 0.22 μm membrane filter is used to remove large EVs, cells, and cellular debris. (II) Dialysis removes free proteins with a typical membrane size of 500 kDa. (III) Finally, the sample is filtered with a 100 nm membrane filter. This technique does not need highly specialized equipment and significantly decreases processing time from about 16 hours for ultracentrifugation to minutes [32]. The second method used to isolate sEVs *via* size is SEC [32]. SEC is a traditional technique comprising a porous gel bead phase and a stationary phase, which regulates the passage of different particle sizes through the column. The advantage of SEC is that it is a relatively fast and cost-effective method and doesn’t affect sEVs integrity since it doesn’t rely on high sheer forces like ultracentrifugation [33]. One of the drawbacks of this approach is that it is low throughput and produces sEVs yields [33].

### Polymer-based precipitation

Several commercial polymer-based isolation kits, such as Exo-spin^™^ (Cambridge Bioscience), Total Exosome Isolation (ThermoFisher Scientific), and ExoQuick^®^ (System Biosciences), are widely used to isolate sEVs from serum, plasma, and cell culture media. This is because these kits require less time and lower centrifugation speeds [34]. These kits work by forming a polymeric layer, such as polyethylene glycol (PEG), around the outer surface of the sEVs. The formation of the polymeric layer around sEVs decreases the centrifugation speed 100 times compared to ultracentrifugation (e.g., 1,000 *g* for polymer-based precipitation *vs*. 100,000 *g* for ultracentrifugation) [34].

In 2019, Patel et al. [35] studied the impact of sEVs isolation kits, including 101Bio, Invitrogen, MagCapture, iZON, and conventional ultracentrifugation, on sEVs size, zeta potential, yield, RNA/protein quality, and marker expression. The average size of sEVs isolated by 101Bio, Invitrogen, MagCapture, iZON, and conventional ultracentrifugation was 114.93 nm ± 11.92 nm, 182 nm ± 13.92 nm, 132.7 nm ± 2.65 nm, 134.13 nm ± 6.46 nm, and 120.07 nm ± 8.26 nm, respectively [35]. Moreover, there was a difference in sEVs yield between the isolation methods, where Invitrogen kits produced the highest yield. In contrast, the MagCapture isolation method yielded the least [35]. These results indicate the variability in different polymer-based methods and demonstrate the importance of optimizing this step based on the desired application.

Isolation methods, such as ultracentrifugation, size-based isolation, and polymer-based precipitation, cannot isolate EVs derived from a specific cell type, such as neuronal EVs, but rather separate them from other components of the sample, such as cells, debris, and apoptotic bodies. Therefore, researchers aiming to utilize sEVs to diagnose NDDs might need to apply additional isolation methods to target brain-derived sEVs and differentiate them from other EVs. For example, Mustapic et al. [36] utilized a two-step technique to isolate neuronal-derived sEVs from blood samples: (I) sEVs were isolated using a commercial isolation kit, namely ExoQuick. (II) Next, they utilized immunoprecipitation (discussed in the next section) to enrich neuronal-derived sEVs by targeting the L1 cell adhesion molecule (L1CAM) *via* biotinylated antibodies. The following discussion will highlight immunoprecipitation isolation methods and the enrichment of brain-derived sEVs from biological samples.

### Immunoprecipitation

Immunoprecipitation is a highly specific isolation method that separates sEVs from EVs and other biological constituents using antibodies to target proteins expressed on the surface of sEVs. Selecting proteins to target for successful isolation relies on several factors, such as the cell of origin, protein concentration, and orientation on the outer surface. For example, neural cell adhesion molecule (NCAM) and L1CAM are often targeted to isolate neuronal sEVs [37], 2’,3’-cyclic nucleotide-3’-phosphodiesterase (CNPase) has been used to isolate oligodendroglial sEVs [38], and glutamate aspartate transporter (GLAST) has been used for astrocyte-derived sEVs [39].

**Figure 3.**
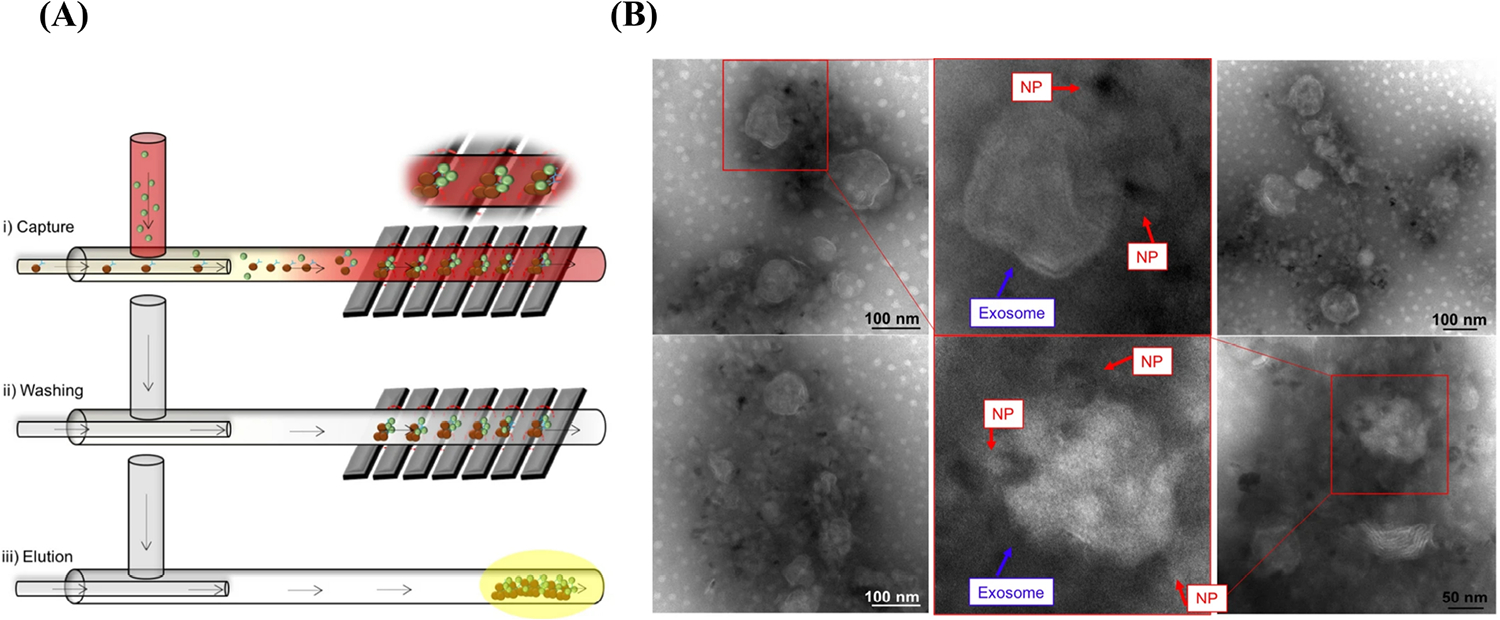
Neuronal sEVs captured by using a microfluidic strategy. (A) Schematic representation of sEVs isolated *via* a microfluidic device [40]; (B) TEM images show sEVs captured by Fe_3_O_4_ NPs conjugated with anti-CD9 [40] *Note*. Figure 3A was reprinted from “Isolation of exosomes from whole blood by a new microfluidic device: proof of concept application in the diagnosis and monitoring of pancreatic cancer,” by Sancho-Albero M, Sebastián V, Sesé J, Pazo-Cid R, Mendoza G, Arruebo M, et al. J Nanobiotechnology. 2020;18:150 (https://doi.org/10.1186/s12951-020-00701-7). CC BY; Figure 3B was reprinted from “Isolation of exosomes from whole blood by a new microfluidic device: proof of concept application in the diagnosis and monitoring of pancreatic cancer,” by Sancho-Albero M, Sebastián V, Sesé J, Pazo-Cid R, Mendoza G, Arruebo M, et al. J Nanobiotechnology. 2020;18:150 (https://doi.org/10.1186/s12951-020-00701-7). CC BY.

Recently, several novel isolation techniques and commercial kits have been designed based on sEVs protein markers antibody affinity binding, including microfluidics and the magnetic bead-based isolation kits (e.g., MagCapture) [40]. A recent study showed the isolation of sEVs from whole blood by microfluidic channels [40]. As shown in Figure 3A, sEVs were captured through magnetic nanoparticles [NPs; iron oxide (Fe_3_O_4_) NPs] conjugated with CD9 antibodies, and sEVs-NP complexes were entrapped *via* the application of a high magnetic field generated by attaching a permanent magnet to the walls of the microchannel [40]. The microfluidic device designed in this study shows a promising novel, low-cost, fast, and high-purity sEVs isolation technique. The transmission electron microscope (TEM) images in Figure 3B show that the microfluidic device successfully captured sEVs from whole blood samples. Moreover, functionalizing Fe_3_O_4_ NPs with anti-L1CAM instead of anti-CD9 might result in a promising tool for researchers interested in the isolation of neuronal-derived sEVs from biological samples.

Although isolation techniques that rely on sEVs protein markers antibody affinity binding are highly specific and increase purity, these techniques have several drawbacks, which include low sEVs yields and the high cost of antibodies. This restricts these methods to small sample volumes, and there are limitations with respect to scale-up. Currently, no isolation technique has been perfected, specifically within the context of clinical translation. The isolation methods are detailed in Table 1, highlighting each technique’s primary advantages and disadvantages. The selection of an appropriate isolation method heavily relies on the sample type, downstream application, and the degree of purity required. Research is ongoing to improve isolation and ultrasensitive detection toward enhancing NDD diagnosis *via* peripheral sEVs. For instance, nanomaterial-based approaches have shown potential as a promising tool to isolate cell specific-derived sEVs, such as brain-derived sEVs from biological samples [41, 42]. This is due to the fact that NPs can be functionalized with ligands, such as DNA, proteins, enzymes, and antibodies [43]. As discussed, and shown in Figure 3A, Sancho-Albero et al. [40] captured sEVs in their microfluidic device using Fe_3_O_4_ NPs functionalized with anti-CD9. Readers may refer to reference [30] to learn more about sEVs isolation.

## AD prevalence and pathology

AD is the most common NDD and accounts for more than 60% of worldwide dementia cases in aged people [44]. Dementia is related to a group of symptoms associated with cognitive decline and impairment in daily activities, which often inhibit patients from living independently [8, 44]. AD is considered a complicated multifactorial disease, and the primary cause of the disease remains unclear. However, there are a few hypotheses that have been suggested as hallmarks of AD, such as the extracellular accumulation of Aβ plaques and the intracellular aggregation of hyperphosphorylated tau (P-tau) proteins, which compromises the ability of neurons to communicate with one another [45].

In the brain of patients without AD, an integral plasma membrane protein called amyloid precursor protein (APP) is cleaved by α-secretase into soluble APP-alpha (sAPPα), and a membrane-bound 83 amino acid C-terminal fragment (CTF), known as CTF83 [46]. The latter is further cut by γ-secretase producing and secreting a soluble peptide, known as P3 [46]. Whereas in individuals with AD, instead of α-secretase, APP is cleaved by another enzyme called β-secretase into truncated soluble APP-beta (sAPPβ) and membrane-bound CTF99 is further cleaved by γ-secretase producing insoluble forms of Aβ, such as Aβ40 and Aβ42 [46]. The insoluble Aβ binds together and produces extracellular Aβ plaques. This likely explains why β-secretase and γ-secretase are primary targets in drug discovery for AD, as they have a major role in the synthesis and secretion of Aβ40 and Aβ42. Another hallmark of AD is the intracellular accumulation of neurofibrillary tangles (NFTs) made up of P-tau [47]. Under normal conditions, tau acts as building blocks for microtubules that facilitate intracellular transport [47]. Abnormal hyperphosphorylation of tau results in the dissociation of tau molecules from microtubules and eventually they form intracellular NFTs [47]. However, the relationship between Aβ and tau pathologies is not yet clear. One hypothesis suggests that Aβ and tau pathologies begin independently but then function synergistically to cause synaptic dysfunction and neuronal apoptosis. For example, Jackson et al. [48] identified that upregulating tau in a mouse model of AD increased the dystrophic neurite number and size of Aβ-plaques.

Several therapeutic molecules have shown promising results in decreasing the concentration of Aβ40, Aβ42, and tau in preclinical trials. However, when they reached clinical trials, results were disappointing and some even led to more damage than cure [49, 50]. This could be explained by the fact that these treatments were studied on mild-to-moderate individuals with AD, and by the time these individuals reached this stage, significant irreversible damage had already been done to neuronal cells as a result of the accumulation of Aβ plaques and NFTs. In fact, the pathophysiologic process of AD begins 10 years to 20 years before symptoms start to appear [51]. Therefore, efforts have switched to focus on earlier phases of the disease to intervene before irreversible damage to the brain and memory loss occurs [51]. The presymptomatic stage seems to be an optimum period for treating AD as in this stage as there is evidence for an imbalance between the production and the clearance of Aβ and P-tau; however, it has not yet produced irreversible damage to the brain [51]. Therefore, treating AD effectively necessitates early detection and a combination of new diagnostic techniques and biomarkers, such as sEVs-based approaches, are critically needed. Several researchers have found that sEVs play a key role in the pathogenesis of AD since they act as vehicles that carry pathogenic proteins, such as Aβ and tau, between cells in the brain [52]. In addition, evidence for their transport across the BBB is of particular interest to access these biomarker repositories that may not otherwise be obtainable in the peripheral circulation. Thus, the potential to isolate brain-derived sEVs from the peripheral circulation and characterize their content (e.g., proteins, lipids, or RNA) could yield a new method to diagnose AD at the pre-symptomatic stage and assist therapeutic candidates in achieving their potential as cures.

## sEVs as sources of biomarkers for AD

**Figure 4.**
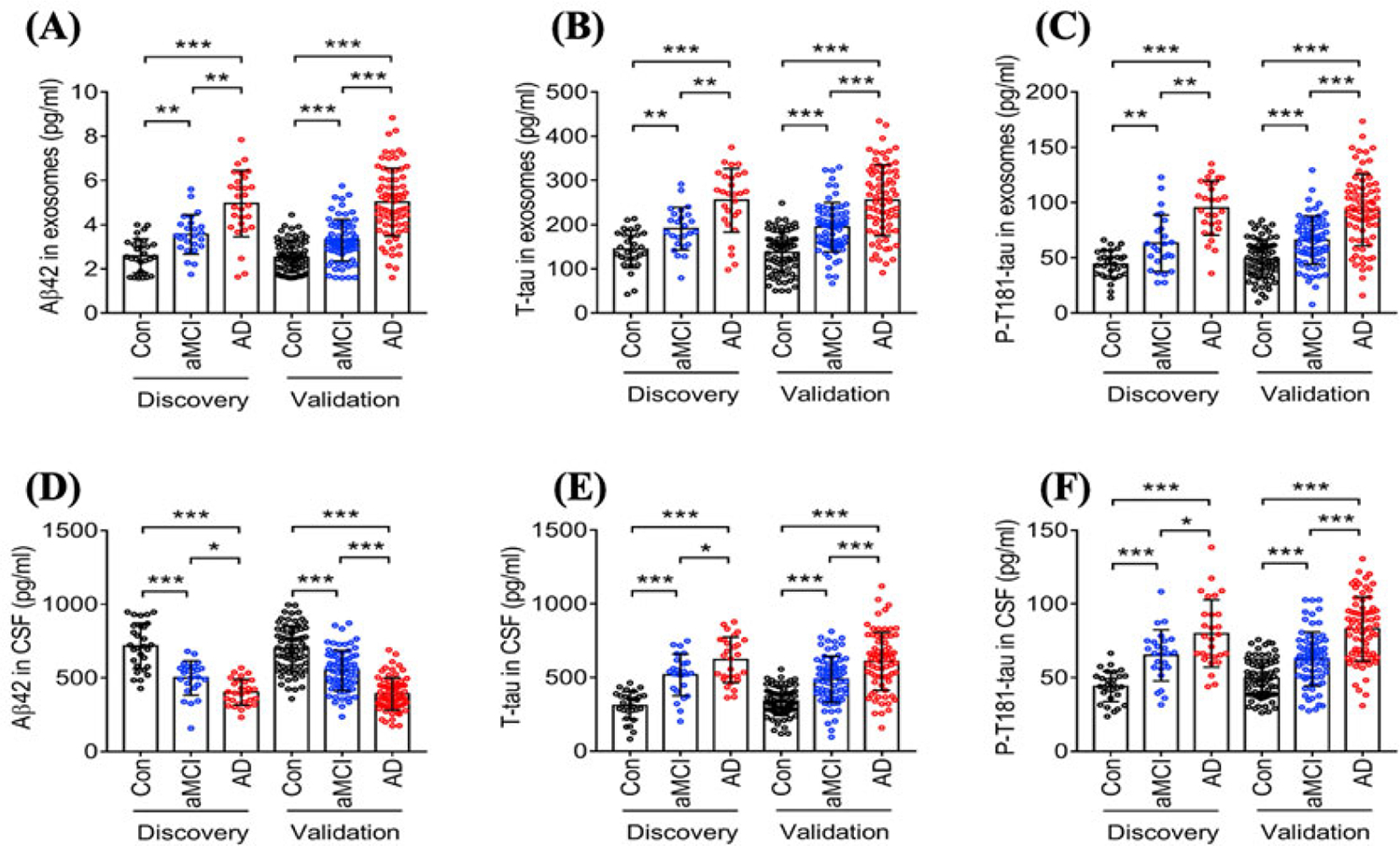
Statistical analysis studying the difference in the concentration of different AD biomarkers in sEVs collected from blood samples of AD patients, patients in the aMCI, and healthy controls in two data sets (discovery and validation). (A) Aβ42 concentrations in neuronal-derived sEVs isolated from the blood of AD patients, patients in the aMCI stage, and healthy controls; (B) T-tau concentrations in neuronal-derived sEVs isolated from the blood of AD patients, patients in the aMCI stage, and healthy controls; (C) P-T181-tau concentrations in neuronal-derived sEVs isolated from the blood of AD patients, patients in the aMCI stage, and healthy controls; (D) Aβ42 concentrations in neuronal-derived sEVs isolated from CSF of AD patients, patients in the aMCI stage, and healthy controls; (E) T-tau concentrations in neuronal-derived sEVs isolated from CSF of AD patients, patients in the aMCI stage, and healthy controls; (F) P-T181-tau concentrations in neuronal-derived sEVs isolated from CSF of AD patients, patients in the aMCI stage, and healthy controls [56]. Con: controls *Note*. Reprinted from “Concordance between the assessment of Aβ42, T-tau, and P-T181-tau in peripheral blood neuronal-derived exosomes and cerebrospinal fluid,” by Jia L, Qiu Q, Zhang H, Chu L, Du Y, Zhang J, et al. Alzheimers Dement. 2019;15:1071–80 (https://doi.org/10.1016/j.jalz.2019.05.002). CC BY-NC-ND.

Researchers strive to identify biochemical markers since they have the potential to provide critical insight into disease pathogenesis, monitor response to treatments, and accelerate new diagnostic tools that can detect diseases in their early stages to enable more successful interventions. In fact, several studies have shown that Aβ1−42, total tau (T-tau), and P-tau present in the CSF have the potential to provide critical diagnostic information in the early stages of AD [53, 54]. However, CSF collection through lumbar punctures is highly invasive and, therefore, not well-suited for routine testing [55]. Thus, identifying blood-based biomarkers and techniques for reliable and cost-effective detection could open a new source of possibilities to screen for AD at these critical early stages. Due to the fact that EVs, such as sEVs, can be formed and secreted from brain cells and pass through the BBB into the circulation, mounting attention has focused on these nano-vesicles as repositories for AD-related biomarkers due to their ability to reflect processes in the brain [55]. Jia et al. (2019) [56] studied the diagnostic ability of blood circulating sEVs proteins, including Aβ42, T-tau, and P-T181-tau in amnestic mild cognitive impairment (aMCI) and individuals with AD and correlated them with concentrations in CSF. The study was conducted in two stages: (I) discovery, which included 28 AD patients, 25 aMCI patients, and 29 controls; (II) validation, to confirm the results of the discovery stage. Stage II included 73 AD, 71 aMCI, and 72 controls. The authors found that the concentration of sEVs’ Aβ42, T-tau, and P-T181-tau in samples from the AD group was significantly higher than samples from aMCI and control groups, as shown in Figure 4 [56]. Furthermore, the concentration of sEVs’ Aβ42, T-tau, and P-T181-tau in samples from aMCI patients were lower compared to samples from AD patients but higher than in the control group. Results were similar in both the discovery and validation stages. In other words, by using the concentration of sEVs’ Aβ42, T-tau, and P-T181-tau, researchers were not only able to detect the disease but also distinguish between individuals at different stages of the disease. The authors contend that sEVs’ Aβ42, T-tau, and P-T181-tau have the capacity to diagnose AD similar to those present in CSF since there was a high correlation between sEVs biomarkers in the blood and CSF [56]. A year later, Jia et al. [57] found that the expression of sEVs synaptic proteins, including neurogranin (NRGN), growth-associated protein 43 (GAP43), synaptotagmin 1, and synaptosome-associated protein 25 (SNAP25), was lower in AD patients than in healthy controls. The authors also observed a slight decrease in the expression of these sEVs synaptic proteins in preclinical AD groups compared to healthy controls. Furthermore, they stated that by combining the expression of these sEVs biomarkers in preclinical AD groups, they were able to predict the disease 5 years to 7 years before cognitive impairment starts to appear [57].

A case-control study was done by Fiandaca et al. [58] showing that blood-derived sEVs biomarkers, including P-S396-tau, P-T181-tau, and Aβ1–42 were able to detect AD up to 10 years prior to clinical onset. This is likely because the concentration of the sEVs biomarkers from AD blood samples was highly upregulated compared to control samples. Another study examined the concentration of sEVs’ Aβ1–42, P-S396-tau, and P-T181-tau in 4 groups: (I) individuals with mild to moderate AD, (II) individuals with MCI, (III) individuals who shifted from MCI to AD within the past 36 months, and (IV) controls [59]. The authors found that the concentration of Aβ1–42, P-S396-tau, and P-T181-tau increased significantly in individuals with AD and patients who transitioned from MCI to AD [59]. This indicates that this test can distinguish individuals with different stages of the disease and predict individuals who will shift from MCI into AD. Overall, these results provide proof of concept that sEVs have the potential to detect AD in early stages. The expression of protein-based biomarkers for AD found in sEVs across several recent studies is summarized in Table 2.

The critical need for new AD diagnostic strategies has fueled growing research into the utilization of brain-derived blood circulating sEVs, new sources for biomarkers. This interest has ignited the hope of improving current diagnostic strategies in a way that can be less invasive and more sensitive to detect the disease in its early stages and assist the discovery of new therapeutic molecules to improve prognosis and address the complexities of AD, with better clinical outcomes compared to current interventions.

## PD prevalence and pathology

PD is a debilitating age-related NDD and movement disorder. PD is second only to AD in NDD prevalence, affecting roughly 1–2 individuals per 1,000 people [60]. The onset of the disease usually occurs between the ages of 65–70 with less than 5% of cases occurring before 40 [60]. Most cases fall under the idiopathic classification since there is often no obvious cause. However, almost all early-onset cases are viewed as genetic variants of the disease, though these only make up roughly 5–10% of known cases. Monogenetic or familial forms of PD are rare in the overall population with select groups showing higher incidence rates [60]. PD proves to be very difficult to diagnose, particularly at early states, though even roughly a quarter of clinically diagnosed cases are reclassified postmortem [61]. This is likely due to the varying presentation seen from case to case, and the continued reliance on motor symptom observation as the gold standard for PD diagnosis.

While the precise cause of PD is still unclear, some hypothesize that certain risk factors affect the homeostasis of neurons in the substantia nigra pars compacta (SNpc), leading to the initiation and progression of neurodegeneration [62]. Many of these risk factors cause an increased state of oxidative stress (OS), which is an imbalance between the assembly and breakdown of reactive oxygen species (ROS). The dynamic balance between the production and clearance of ROS is more relevant in PD than AD. This is due in large part to the mechanism of formation of Lewy bodies and neurites in the brain, which can be induced by OS [63]. OS can affect the expression of various miRNAs, while inversely, miRNAs can, in turn, regulate the genes involved in the mediation of OS. Despite the potential protection offered by crosstalk between sEVs miRNAs and OS in PD, this crosstalk remains equally capable of doing harm to the same system it offers protection. For example, the release of miR-34a from astrocytes *via* sEVs has been shown by PD models to enhance the sensitivity of dopaminergic neurons to neurotoxins due to the targeting of B-cell leukemia/lymphoma 2 (Bcl-2) protein [60]. While another study has provided evidence of the alleviation of oxidative stress-induced neuronal apoptosis with the upregulation of the same miR-34a [60]. Evidence is growing to support the pathophysiological role of miRNAs, like miR-34a, in PD. Additionally, the imbalances caused by the accumulation of ROS can lead to the oxidation and subsequent modification of molecules and proteins, such as dopamine (DA) and α-syn, which are necessary for proper cellular function [64]. These modified proteins and molecules may be used as diagnostic biomarkers for PD, given their role in pathogenesis and progression.

α-Syn is a neuronal protein present in high concentrations at pre-synaptic nerve terminals [65]. Though its exact functions remain poorly documented, several roles have been suggested. First, is its involvement in the regulation of neurotransmitters due to high pre-synaptic concentrations. This is exhibited by restriction in synaptic vesicle mobility, thus decreasing neurotransmitter release and the recycling of synaptic vesicles [66]. Second, is the binding of vesicle-associated membrane protein 2 (VAMP2) to α-syn, which may contribute to the stability of presynaptic soluble *N*-ethylmaleimide-sensitive factor (NSF) attachment protein receptor (SNARE) complexes. This is necessary for α-syn-mediated devitalization of synaptic vesicle recycling. Lastly, α-syn was found to mediate DA synthesis by acting as an inhibitor of tyrosine hydroxylase, which is crucial in individuals with PD due to the loss of dopaminergic neurons as the disease progresses [66].

α-Syn primarily exists in two states within the body, soluble and membrane-bound. These two forms remain at equilibrium with one another under non-pathogenic conditions with the form taken dictating the secondary structure assumed by the protein [65]. Soluble α-syn is normally unstructured and monomeric in nature. Meanwhile, membrane-bound α-syn structurally conforms to an amphipathic α-helix associated with both multimerization and SNARE-complex chaperoning functions. This structural change occurs upon the binding of the protein to the curved membrane of a vesicle [65]. Pathological conformations of α-syn differ from standard physiological conformations. Under pathological conditions, α-syn will form β-sheet structures. These sheet conformations have been connected to the aggregation of α-syn, fibril formation, and the deposition of Lewy bodies [65]. While many believe these β-sheet conformations to be neurotoxic, the exact nature of the toxicity and the primary cause of pathological conformations is still poorly understood [65]. Several mechanisms are believed to play a role in the pathology of α-syn, but the most attributed PD pathophysiology are post-translational modifications. α-syn is prone to multiple types of post-translational modifications (e.g., phosphorylation, glycation, nitration, and oxidation), specifically on the carboxyterminal tail [65]. While the rate of occurrence of post-translational modifications is difficult to gauge, modifications to α-syn that alter the structure and function have a high likelihood of causing oligomers or aggregates to form [66].

While α-syn is a common biomarker associated with PD, the neurotransmitter DA is also of interest due to the loss of DA-producing neurons in the SNpc. DA in the SNpc is stringently regulated to maintain an equilibrium between the functions of synthesis, synaptic vesicle loading, uptake, and catabolic degradation [62]. The catabolism of DA begins with oxidative deamination, which results in the formation of hydrogen peroxide (H_2_O_2_), ammonia, and 3,4-dihydroxyphenylacetaldehyde (DOPAL). Although DOPAL is a standard metabolite of DA catabolism, it is shown to be neurotoxic [47]. Under physiological conditions, DA is metabolized into DOPAL and then further metabolized into 3,4-dihydroxyphenylacetic acid (DOPAC) or 3,4-dihydroxyphenylethanol (DOPET) [62]. However, certain risk factors are believed to cause DOPAL metabolites to stall at this intermediate stage of DA catabolism leading to the creation of an increasingly neurotoxic environment and the eventual death of dopaminergic neurons in the SNpc. DOPAL is an extremely reactive molecule with two functional groups, an aldehyde and a catechol, contributing to its reactivity with proteins [62]. The catechol group is subject to auto-oxidization by reactive quinones, and the aldehyde group is known to cause cellular damage by reacting to generate covalent addition products [67]. The ‘catecholaldehyde hypothesis’ postulates that a neurotoxin specific to DA-producing neurons plays a role in their decline in PD [67]. Masato et al. [62] relate this hypothesis to the α-syn-induced pathology of PD, reflecting on the interactions between DOPAL and α-syn. First, DOPAL is composed of roughly 10% lysine. Due to the reactivity observed between the DOPAL aldehydes and the primary amines of lysine residues, the increased lysine present points to a higher potential for α-syn to be modified by DOPAL [62].

Second, α-syn accounts for approximately 1% of the total soluble protein in the brain and is highly present at pre-synaptic nerve terminals. DOPAL being highly concentrated on the pre-synaptic space increases the potential for interactions with α-syn [62]. Lastly, when in its soluble form, α-syn is a naturally disordered protein with ready access to lysine residues. This greatly increases the chance of chemical alteration of α-syn by DOPAL [62]. Biomarkers, such as DA and α-syn, hold considerable promise for improving PD diagnostics. Since the pathophysiological progression of PD can begin years prior to the onset of symptoms, it is crucial to develop methods that enable the diagnosis at pre- or early stages to intervene more effectively. At these stages, post-translational modifications of key proteins and molecules have not yet led to major disruptions in the homeostatic equilibrium of neurons or the aggregation of certain protein fibrils. The dire need for improved early diagnostic methods opens the door for new and innovative research like that of PD biomarkers and sEVs. As with AD, new evidence has supported the ability of sEVs to aid in improving PD diagnostics due to their role in transporting molecules and proteins from cell-to-cell, and the ability to cross the BBB [64, 68, 69]. The wide range of potential uses for sEVs in both diagnostics and therapeutics offers new hope to those individuals suffering from neurodegenerative conditions.

## sEVs as sources of biomarkers for PD

In addition to their role in transportation, sEVs or exosomes are further understood to aid in nerve regeneration and the maintenance of synaptic plasticity [69]. While specific functions and mechanisms of sEVs and their role in the progression of PD pathogenesis are still being explored, a growing body of evidence suggests sEVs can pass pathogenic proteins and neurotoxic molecules to neighboring healthy cells [70, 71]. In this way, sEVs have been shown to exacerbate the prion-like behavior of certain modified proteins, like α-syn. As these modified proteins are passed from cell-to-cell, a possible correlation between the movement of these modified proteins and PD has been identified [72]. This transmission represents an important avenue of discovery for both the initiation and progression of PD, as well as a therapeutic target. Additionally, this transportation mechanism gives strong credibility to the potential of circulating sEVs as a reliable source of biomarkers for screening purposes.

There are two main roles sEVs are known to play in PD pathogenesis as it relates to α-syn: (I) they act as the principal mediators of α-syn transmission from cell-to-cell; (II) they contribute to non-cell autonomous-mediated neurotoxicity [64]. While sEVs are not the only regulated method of α-syn release and uptake, they have been found to conduce non-cell mediated neurotoxicity and modulate α-syn conveyance over a wider range than most other transmission mechanisms. sEVs-associated α-syn oligomers were determined to increase the potential of cellular uptake, thus causing a greater level of neurotoxicity compared to other non-sEVs-associated α-syn oligomers [64]. For example, Han et al. [73] showed that treating mice with 200 μg of sEVs isolated from PD patient serum-induced α-syn aggregation and DA neuron degeneration.

Since sEVs act as a vehicle for α-syn and carry it between cells in the brain and across the BBB, analyzing circulating sEVs derived from blood has the potential to serve as a promising repository for PD-associated biomarkers to fuel the research into the design of new diagnostic tools for early stage detection [63, 64, 68, 74]. Unfortunately, most of the current testing methods, outside of routine patient observation, rely heavily on imaging systems like computed tomography (CT), magnetic resonance imaging (MRI), positron emission tomography (PET), and single photon emission CT (SPECT) with DA transporter (DAT). While these systems are effective at ruling out the presence of conditions which can mimic PD, they lack the ability to give a conclusive Parkinson’s diagnosis [75]. The few non-imaging methods available are often invasive or require application over longer durations. Two examples are the spinal tap, which procures CSF for analysis, and Levodopa dosing. The characteristics of these diagnostic methods make them not well suited for retrieval of quick diagnostic results or regular use in the tracking of disease progression [76]. For this reason, PD like AD is almost exclusively diagnosed at the symptomatic stage of disease progression. At this stage of progression, irreversible damage has already occurred to the dopaminergic neurons of the SNpc [77]. Thus, researchers strive to identify functional applications of sEVs, especially those circulating in the blood, and to study the ability of these sEVs to mirror the progression of PD in the brain.

For example, Shi et al. [31] studied α-syn from neuronal-derived sEVs isolated from plasma as a biomarker for PD by isolating L1CAM-containing sEVs from the peripheral circulation of 267 individuals with PD and comparing it to the concentration of α-syn from sEVs isolated from 214 healthy controls. They found that the concentration of sEVs α-syn was significantly increased in samples obtained from individuals with PD compared to controls [31]. The concentration of sEVs α-syn was also highly correlated with the severity of the disease [31]. Another paper studied the ability of two plasma neural-derived sEVs biomarkers, α-syn and protein deglycase DJ-1 (DJ-1), to diagnose PD in its early stages [78]. It was found that the concentration of both α-syn and DJ-1 from sEVs obtained from individuals in early- and advanced-stage PD plasma was significantly upregulated compared to that obtained from the control group [78]. However, the biomarkers could not distinguish patients in the early stage of the disease from patients with advanced PD. There was a significant correlation between the concentration of the two plasma neural-derived sEVs biomarkers from individuals with PD, whereas this correlation was not in sEVs obtained from the controls [78].

**Figure 5.**
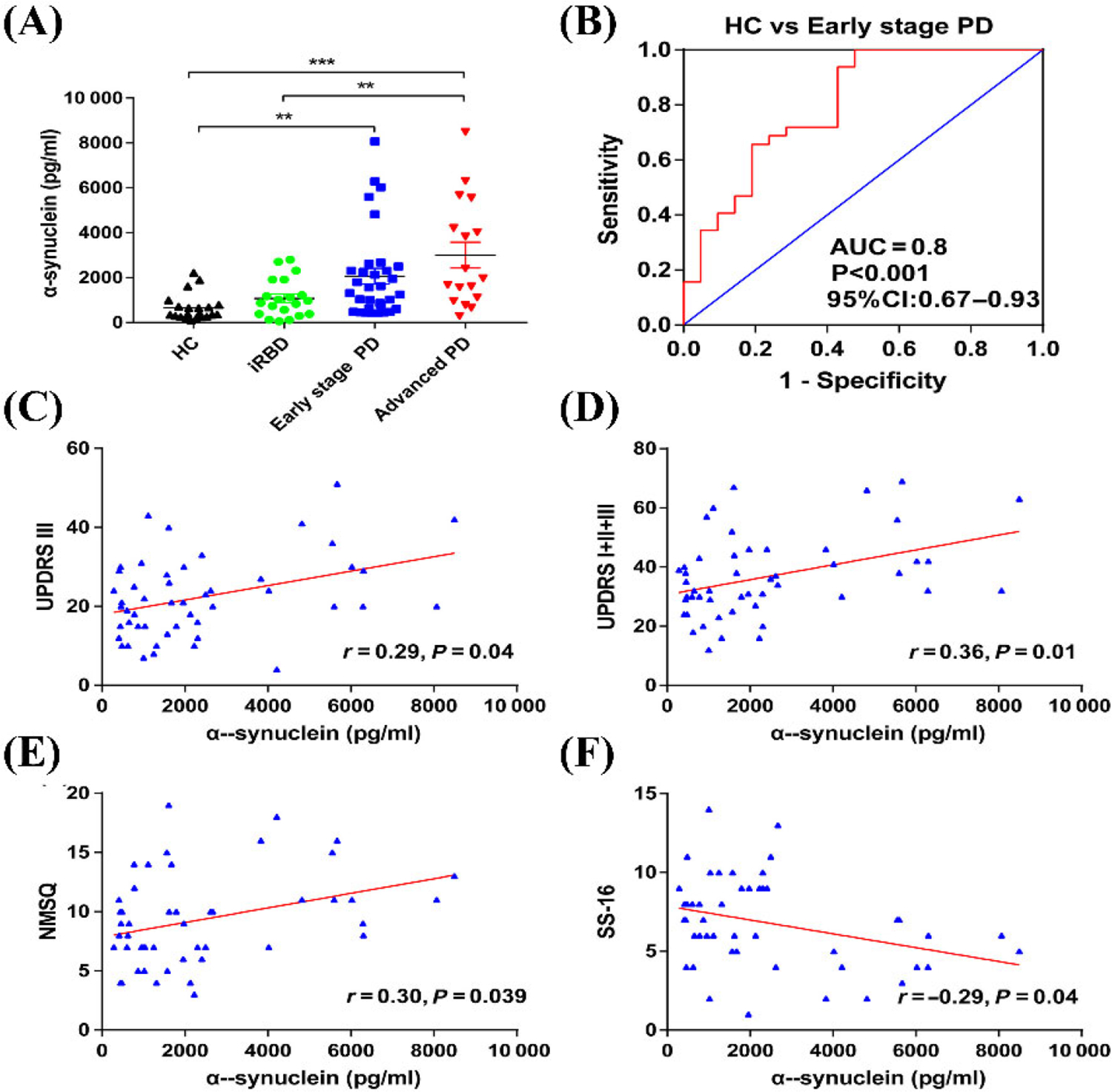
The concentration of α-syn in plasma-derived neuronal sEVs. (A) α-Syn concentrations derived from neuronal sEVs in the plasma samples of healthy controls, individuals with idiopathic rapid eye movement sleep behavior disorder (iRBD), early-stage PD, and advanced PD. Data presented are mean ± standard deviation (SD), ** *P* < 0.001, *** *P* < 0.001. HC: healthy controls; (B) an ROC analysis showed that α-syn could be used to differentiate individuals with early-stage PD and HCs. Area under the curve = 0.8, accuracy = 0.83; (C) the correlation between the concentration of α-syn in plasma neuronal sEVs with Unified PD Rating Scale (UPDRS) III scores; (D) the correlation between the concentration of α-syn in plasma neuronal sEVs with UPDRS (I + II + III) scores; (E) Non-Motor Symptom Questionnaire (NMSQ) scores; (F) 16-item odor identification test from Sniffin’ Sticks (SS-16) scores in patients with PD *Note*. Reprinted with permission from “A longitudinal study on α-syn in plasma neuronal exosomes as a biomarker for Parkinson’s disease development and progression,” by Niu M, Li Y, Li G, Zhou L, Luo N, Yao M, et al. Eur J Neurol. 2020;27:967–74 (https://doi.org/10.1111/ene.14208). © 2020 European Academy of Neurology.

Niu et al. [79] designed a longitudinal and cross-sectional study to characterize the ability of α-syn in plasma neuronal sEVs to be used as a biomarker to diagnose PD in its early stages. Neuronal sEVs were isolated from blood samples of 36 participants with early-stage PD, 17 participants with advanced-stage PD, and 21 healthy participants [79]. The researchers found that neuronal sEVs isolated from blood plasma samples of individuals with PD contained a significantly higher concentration of α-syn compared to controls [79]. The researchers contend that the concentration of sEVs α-syn was able to determine early-stage PD participants from controls with 100% sensitivity and 57.1% specificity, as shown in Figure 5. Furthermore, 18 patients from the early-stage group participated in a follow-up study for an average of 22 months. From this longitudinal study, the authors found that sEVs α-syn was not only a biomarker for the early detection of PD but also showed potential as a prognostic marker [79].

To summarize, sEVs are gaining much attention as a source of biomarkers for diagnosing PD. This disease jeopardizes the quality of life of about 2% to 3% of the worldwide population [80]. Moreover, since sEVs could be utilized to diagnose individuals who do not present clinical symptoms, they can potentially accelerate the discovery of therapeutic strategies for PD.

## sEVs as biomarkers for diverse neurological diseases

HD is a rare, inherited, incurable, and fatal neurodegenerative disorder with a prevalence of 2.71 per 100,000 people worldwide [81]. HD is a progressive disease that causes functional, behavioral, and cognitive decline leading to a devastating impact on patients and their families [82]. HD is primarily caused by the expansion of cytosine-adenine-guanine (CAG) trinucleotide repeats in the huntingtin (HTT) protein-coding gene *HTT* gene that leads to the formation of a mutant form of mHTT containing an abnormal number of glutamine amino acid repeats, known as polyglutamine (polyQ) in its amino terminus (N-terminus) [82]. Yet, no therapies are available to slow down the progression or treat HD [82]. It is still unclear whether sEVs have a role in the pathogenesis of the disease or act as a vehicle to transmit the mutated proteins between brain cells. However, Wang et al. [83] studied the correlation between the altered genes in HD and the protein content of sEVs by screening several databases, namely Exosome ProteinDB and HD Perturbation. It was determined that sEVs protein contents intersected with the HD dataset, which might indicate that sEVs have a role in the disease [83]. Another study found that injecting the ventricle of newborn mice with sEVs derived from HD patients caused the appearance of an HD phenotype and affected cognitive and motor functions [84]. This data may support the hypothesis that sEVs carry mHTT and have a role in the spread of the disease. Although the association between sEVs and the propagation of HD will not be confirmed until it is observed in further *in vitro* or *in vivo*, the studies above indicate sEVs may play some role in HD progression and should therefore be considered as biomarker repositories using similar approaches taken by AD and PD researchers.

MSA is another rare NDD [85]. Like PD, MSA comes under the umbrella of α-synucleinopathy, characterized by the abnormal accumulation of misfolded forms of α-syn [85]. The main hallmark of the disease is glial cytoplasmic inclusions, mainly in oligodendrocytes, that are predominantly made up of α-syn aggregates [85]. Patients with MSA have motor and non-motor symptoms, such as slow movements, postural tremor, action tremor, pain, and dysphagia [86]. Based on the symptoms at the time of diagnosis, MSA is divided into two phenotypes: MSA-parkinsonian type (MSA-P) and MSA-cerebellar type (MSA-C) [86]. Currently, MSA is diagnosed mainly by clinical examination and neuroimaging [86]. However, MSA can be misdiagnosed as PD, especially in the early stages, since they overlap to some extent in their etiology and symptoms. As such, there is a significant need to identify biomarkers that can not only diagnose this disease but also have the potential to distinguish MSA from PD.

Given that sEVs can act as a carrier for α-syn and interferes with the transmission of proteins between brain cells, sEVs may also be a promising source of biomarkers for MSA. In 2020, Jiang et al. [87] found that the concentration of α-syn in neuron-derived sEVs extracted from patients’ plasma could differentiate MSA from PD patients since the concentration of sEVs α-syn from plasma with MSA was twofold lower than that obtained from PD patients. A year later, the researchers validated the consistency of their diagnostic assay by measuring the α-syn in neuronal-derived sEVs obtained from the serum of an additional 267 samples [37]. Combining the number of samples from the two research studies, the authors measured the sEVs α-syn of 735 participants, which makes this the most extensive study of neuronal-derived sEVs α-syn in MSA and PD to date [37]. The authors contend that the results were congruous to their previous paper and, more importantly, they were able to identify that 14.0 ng/L sEVs α-syn is a threshold concentration for PD, and this sEVs α-syn concentration was able to consistently differentiate MSA from PD [37]. However, the concentration of neuronally derived sEVs (L1CAM-immunoprecipitated) was not investigated in each group; thus, it was not clear whether the difference in the sEVs α-syn concentration is mainly driven by the decrease in the number of the L1CAM-positive sEVs in the plasma or the concentration of α-syn protein molecules that are carried into each sEV. In this regard, Yu et al. [38] studied the impact of the occurrence of α-syn aggregation on the secretion mechanism of sEVs from oligodendrocyte cells. The researchers targeted oligodendroglial sEVs rather than neuronal ones because MSA-associated α-syn predominantly accumulates in oligodendrocytes [38]. They found from both *in vitro* and *in vivo* studies that the increase in the presence of α-syn aggregation was correlated with a significant decrease in the release of sEVs in MSA [38]. The findings of Yu et al. [38] could explain the reduction of sEVs α-syn levels in human samples from individuals with MSA.

Overall, sEVs and their content in the peripheral circulation can potentially serve as a promising tool to detect NDDs with a focus on those mentioned above, namely AD, PD, HD, and MSA, in their early stage and non-invasively. While no studies have validated biomarkers derived from sEVs from HD and MSA, the potential for their use in AD and PD holds excellent promise for upcoming developments in this area. However, it is notable that the overlap between the symptoms of these NDDs leads to difficulties in their diagnosis, notwithstanding the differential between symptom manifestation at a clinically relevant level and irreversible changes at the physiological level. Thus, significant efforts have been made to analyze sEVs proteins and their ability to differentiate between NDDs to diagnose early and decrease misdiagnosis events in the clinic. However, the heterogeneity and low concentration of sEVs in blood circulation, particularly compared to the CSF, necessitate the use of highly sensitive and reliable detection techniques that can encounter the limitation of conventional methods, such as western blot and enzyme-linked immunosorbent assay (ELISA). In addition, clinical labs are not well suited to the lengthy processing steps needed to isolate sEVs for analysis. As such, the following discussion will provide a brief overview of biosensor designs using new and promising technologies to detect neuronal sEVs and their constituents from blood.

## Biosensors to detect sEVs in NDDs

From the discussion above, it is clear that employing sEVs from blood circulation as a biomarker for NDDs is receiving attention and being studied more intensively. It is worth noting that most studies mentioned above highly relied on the ELISA approach to detect and quantify neuronal sEVs and their contents. However, the number of sEVs required for ELISA is relatively large, with a limit of detection (LOD) of about 107 particles/μL (depending on the protein concentrations present) [88, 89]. In addition, detection *via* ELISA necessitates lengthy pre-processing steps and isolation techniques. Therefore, there is still a need for highly effective and sensitive quantitative methods to detect neuronal sEVs in blood circulation that are both cost-effective and can produce results rapidly. The need to achieve sensitive sEVs detection has fueled a growing interest in biosensing devices to detect and analyze sEVs from blood directly. This is due to their high accuracy, simple analysis methods, and low cost. Biosensors are analytical tools that can detect and quantify specific biomarkers. Biosensors are composed of two parts: (I) the recognition part, composed of a biological molecule (e.g., antibody, antigen, or DNA) that can specifically interact with a molecule of interest [90]. (II) A transducer to convert the biological interaction between the recognition part and the molecule into a measurable signal [90]. Several methods are used to transduce the biological interaction into signals, such as electrochemical and optical techniques [90]. Biosensors are classified based on the type of biological molecule and transducer used in their fabrication. The following discussion will shed light on several cutting-edge biosensor designs to detect sEVs, focusing on neuronally-derived sEVs.

**Figure 6.**
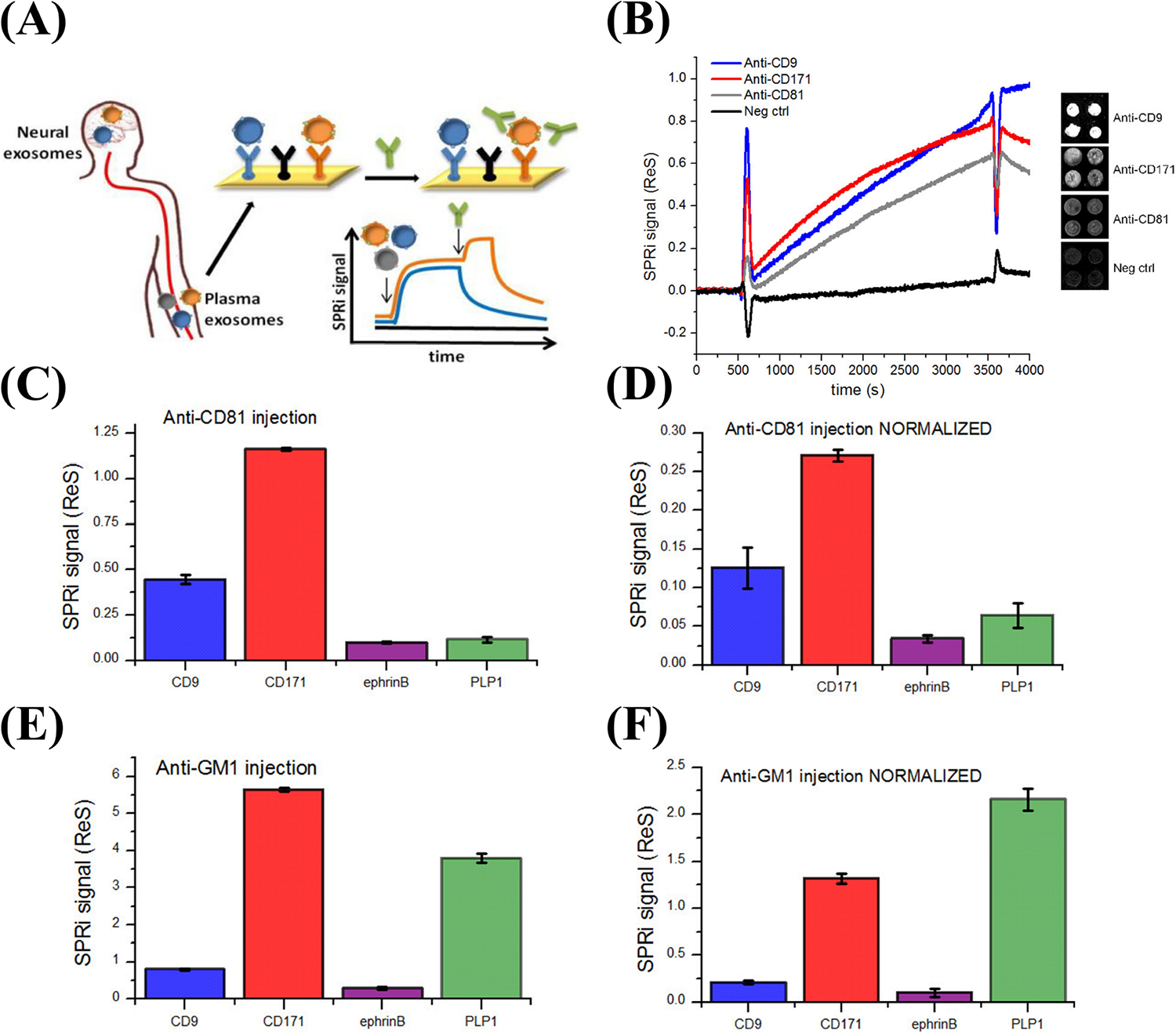
Detection of different subpopulations of sEVs by SPR imaging (SPRi). (A) Illustration of SPRi biosensor to detect neuronal and oligodendroglial sEVs from plasma samples; (B) SPRi sensogram of four different sEV subpopulations. The signal is measured by taking the average of the four spots in each subpopulation and subtracting it from the negative control signals; (C, D) SPRi signal intensity after injection of 200 μl anti-CD81 antibodies at 25 μl/min rate before and after normalization based on the number of sEVs captured on each type of Ab; (E, F) SPRi signal intensity after injection of anti-GM1 antibodies before and after normalization based on the number of sEVs captured on each type of antibody. Neg ctrl: Negative control; SPRi signal (ReS): SPRi signal response; ephrinB: ephrinB is a class of proteins that are highly expressed in neuronal cells; PLP1: proteolipid protein 1 *Note*. Reprinted with permission from “Detection and characterization of different brain-derived subpopulations of plasma exosomes by surface plasmon resonance imaging,” by Picciolini S, Gualerzi A, Vanna R, Sguassero A, Gramatica F, Bedoni M, et al. Anal Chem. 2018;90:8873–80 (https://doi.org/10.1021/acs.analchem.8b00941). © 2018, American Chemical Society.

Picciolini et al. [91] designed a multiplex surface plasmon resonance (SPR) biosensor for applications in NDDs. The authors detected brain-derived sEVs from neuronal and oligodendroglial sEVs from plasma samples. Detection was accomplished using an array of antibodies that were immobilized on a surface of a chip that target protein markers of oligodendroglial and neuronal sEVs [91]. As illustrated in Figure 6, the assay detected sEVs from plasma samples and quantified several protein markers on the outer surface of the detected sEVs, such as CD81 and monosialotetrahexosylganglioside (GM1), by injecting secondary antibodies (e.g., anti-CD81 and anti-ganglioside GM1) into the sensor. This biosensor holds promise for enhancing neuronal sEVs detection and content [91]. Furthermore, since Aβ (AD) and α-syn (PD and MSA) biomarkers are located on the outer surface of sEVs, a minor modification to the Picciolini et al. [91] sensor, such as utilizing anti-Aβ or anti-α-syn instead of anti-CD81 might lead to the use of this sensor as a promising diagnostic assay for AD, PD, and MSA.

Zhou et al. designed a cost-effective, fast, and enzyme-free biosensor that was shown to detect and quantify Aβ42 oligomers in serum samples and sEVs, with a LOD of 20 pmol/L [92]. The sensor comprised of a hairpin probe (H), two different FAM-labelled replacement probes (R1 and R2), and graphene oxide (GO) [92]. Aβ42 oligomers were detected as follows: (I) after adding the sample to the sensor, probe H recognizes Aβ42 oligomers and spreads its stem-loop structure. (II) This alteration in the structure of probe H allows the free single-stranded DNA (ssDNA) R1 and R2 to hybridize with the two ends of probe H and release Aβ42 oligomers from probe H [92]. (III) The released Aβ42 oligomers can interact with probe H to start the same circular interaction again. The role of GO in this sensor was to decrease the background sensor by quenching the excess fluorescence produced by the ssDNA R1 and R2 probes [92]. Due to this biosensor’s high accuracy and precision in detecting Aβ42 oligomers, it can be translated into an effective tool to detect the concentration of Aβ42 oligomers from the plasma of patients in the presymptomatic stage of the disease. Overall, the attractive properties of biosensors, together with the promising results of sEVs as a biomarker for NDDs, could be a solution to screen and diagnose AD, PD, HD, and MSA in their early stage and to overcome the drawbacks of the conventional diagnostic methods in the clinics, such as neuroimaging and CSF analysis.

## Conclusions

NDDs are broadly characterized by the accumulation of a specific type of misfolded protein, which results in the death of neurons in various brain regions. Currently, NDDs are diagnosed by different imaging tools, CSF analysis, and assessments by healthcare practitioners. However, these diagnostic methods can be expensive, invasive, subjective, and are generally unable to detect NDDs in their early stages. The drawbacks of current NDD diagnostic tools, therefore, necessitate the need to develop a new generation of non-invasive and highly accurate techniques that can detect these multifactorial diseases in their early stages to facilitate timely interventions and research on effective therapeutics. Since brain-derived blood circulating sEVs act as carriers for NDD-associated pathological proteins, such as tau, Aβ, α-syn, and mHTT, they are ideal repositories for biomarkers that might provide an accessible avenue to diagnose NDDs in their early stage. This review summarizes sEVs biogenesis, the advantages, and disadvantages of different sEVs isolation techniques, and provides a brief overview of the recent biosensor designs that have the potential to overcome the limitations of the conventional sEVs detection methods. Moreover, the review covers the potential role of sEVs in diagnosing four major NDDs, namely AD, PD, HD, and MSA.

Although significant efforts have been made in applying sEVs as a biomarker for NDDs over the past ten years, several challenges still need to be solved before clinical translation. The following summarizes current gaps in the field and suggested directions to explore the potential application of peripheral circulating sEVs as a novel biomarker for NDDs:

- There is still a lack of standardized sEVs isolation protocols that can consistently isolate with high purity and yield. Thus, integrating new technologies, such as nanomaterials-based approaches and microfluidics, will open the floodgates of possibilities to design efficient and ultrasensitive isolation methods that will help identify new sEVs biomarkers and enhance NDDs diagnosis *via* peripheral sEVs.
- Since sEVs are secreted from every cell in the body, differentiating a subgroup of interest from a mixed population is a significant technical challenge. To this end, there is a critical need to profile the sEVs proteome to enhance the ability to differentiate between sEVs and discover their cell of origin. This will enhance the identification of new sEVs-associated biomarkers and, with respect to NDDs, study the impact of each brain cell type (e.g., astrocytes, oligodendrocytes, and neurons). This new avenue of research holds promise to unravel the complex pathology of NDDs and design new diagnostic strategies to detect NDDs in the presymptomatic stage.
- To identify diagnostic biomarkers for several NDDs, especially rare diseases like HD and MSA, is the lack of sufficient samples with validated diagnoses. This necessitates interdisciplinary methods and close collaborations between community members, healthcare providers, and researchers. The continuity of these types of collaborations would be useful in the development of accessible biobanks, including samples from patients with less prevalent NDDs.

## Figures and Tables

**Table 1.** Summary of common sEV isolation techniques

Isolation method	Advantages	Disadvantages
Ultracentrifugation	Well-established Widely used Simple protocol	Multi-step process Time-consuming Special equipment required Low yields Isolated sEVs could be contaminated with other EVs
Differential filtration	Fast, convenient, and low-cost Doesn’t need special equipment	sEVs loss due to the entrapment of the sEVs in the filters Cross-contamination with other EVs
SEC	Fast, convenient, and low-cost Yields high purity sample	Typically, needs to be combined with another method
Polymer-based precipitation	Easy protocol High yield Low-cost method Doesn’t need special equipment	Cross-contamination with protein aggregates and other EVs Isolated sEVs could be contaminated with the polymer which might affect sEVs characterization, such as zeta potential, and integrity
Immunoprecipitation	High purity Doesn’t need special equipment Based on antigen-antibody interactions, which can discriminate between sEV sub-types and isolate particles of interest	Relies on high-cost antibodies Low yields
Microfluidic separation	Doesn’t need special equipment once fabricated Fast and cost-effective	Low yields Complex designs/fabrication

**Table 2.** Summary of potential sEV-associated protein biomarkers for AD

AD sEVs protein biomarkers	Expression	References
Aβ42	↑	[39, 56, 58, 59]
P-T181-tau	↑	[56, 58]
P-S396-tau	↑	[39, 56, 58, 59]
T-tau	↑	[39, 58, 59]
β-site APP cleaving enzyme 1 (BACE 1)	↑	[39]
γ-Secretase	↑	[39]
sAPPβ	↑	[39]
sAPPα	↑	[39]
Glial-derived neurotrophic factor (GDNF)	↓	[39]
NRGN	↓	[57, 59]
Repressor element 1-silencing transcription factor (REST)	↓	[59]
GAP43	↓	[57]
Synaptotagmin 1	↓	[57]
SNAP25	↓	[57]

↑: the expression of the protein is higher in sEVs collected from samples of AD patients compared to that collected from controls;

↓: the expression of the protein is lower in sEVs collected from samples of AD patients compared to that collected from controls

## Abbreviations

AD: Alzheimer’s disease
aMCI: amnestic mild cognitive impairment
APP: amyloid precursor protein
Aβ: amyloid-β
BBB: blood-brain barrier
CD: cluster of differentiation
CNS: central nervous system
CSF: cerebrospinal fluid
DA: dopamine
DOPAL: 3,4-dihydroxyphenylacetaldehyde
EE: early endosome
ELISA: enzyme-linked immunosorbent assay
EVs: extracellular vesicles
H: hairpin probe
HD: Huntington’s disease
ILVs: intraluminal vesicles
L1CAM: L1 cell adhesion molecule
LEs: late endosomes
mHTT: mutant huntingtin protein
miRNA: microRNA
MSA: multiple system atrophy
MVB: multivesicular body
NDDs: neurodegenerative diseases
NFTs: neurofibrillary tangles
NPs: nanoparticles
OS: oxidative stress
PD: Parkinson’s disease
P-tau: hyperphosphorylated tau
ROS: reactive oxygen species
SEC: size exclusion chromatography
sEVs: small extracellular vesicles
SNpc: substantia nigra pars compacta
SPRi: surface plasmon resonance imaging
T-tau: total tau
α-syn: α-synuclein
